# Revamping Stability: Tibial Tuberosity Transfer and Medial Patello-Femoral Ligament Reconstruction for Patella Alta and Recurrent Dislocation

**DOI:** 10.7759/cureus.54891

**Published:** 2024-02-25

**Authors:** Rahul Singh, Amit Saoji, Anmol Suneja, Saksham Goyal, Sachin Goel

**Affiliations:** 1 Department of Orthopaedics, Jawaharlal Nehru Medical College, Datta Meghe Institute of Higher Education and Research, Wardha, IND

**Keywords:** instability, osteoarthritis, tibial tubercle osteotomy, magnetic resonance imaging, medial patellofemoral ligament, tibial tubercle to trochlear groove distance

## Abstract

This case report details the clinical evaluation, imaging findings, and surgical management of a 17-year-old female with a two-year history of persistent knee pain and recurrent patellar dislocations. Despite the absence of traumatic injury, the patient exhibited significant anatomical abnormalities, including a laterally dislocated patella, shallow trochlear groove, increased tibial tuberosity to trochlear groove (TT-TG) distance, and patella alta by calculating Insall-Salvati ratio. The Insall-Salvati ratio is a radiographic measurement used to assess the position of the patella within the knee joint. It is calculated by dividing the length of the patellar tendon (from the lower pole of the patella to its insertion on the tibial tubercle) by the length of the patella itself (from its superior to inferior pole). This ratio is commonly used in the evaluation of patellar tracking disorders and patellar instability. Typically, a ratio greater than 1.2 is considered indicative of patella alta (high-riding patella), while a ratio less than 0.8 suggests patella baja (low-riding patella). The surgical intervention involved a tibial tuberosity osteotomy (TTO), distalization, and medial patellofemoral ligament (MPFL) reconstruction using the gracilis tendon, resulting in successful realignment as confirmed by postoperative imaging. A postoperative rehabilitation program, including physical therapy and pain management, was initiated to optimize recovery and enhance quadriceps strength and proprioception. This case underscores the importance of a comprehensive surgical approach in addressing recurrent patellar dislocation associated with complex anatomical variations, providing insights into effective management strategies for similar cases.

## Introduction

Young individuals, particularly females in their twenties and thirties, are the most affected by recurrent patellofemoral instability [1]. The loss of the medial patellofemoral ligament (MPFL), a crucial soft tissue that gives strength that provides 50% to 60% of the medial restraining force to a lateral dislocation, is a significant contributor to this syndrome [2]. The recurrence rate after a first dislocation varies significantly between studies (13%-40%) and is much greater in persons with proven MPFL injuries diagnosed with MRI. [2]. High-energy trauma following the primary injury, weaker vastus medialis oblique (VMO) muscles, and obesity are risk factors [3]. Recurrent dislocations frequently result in significant soft tissue and joint structural damage, particularly to the articular cartilage of the patella surface, lateral femoral condyle, and trochlea. Instability is classified by Dejour et al. into 'major,' 'objective,' and 'potential' types [4]. Traumatic cartilage damage prevalence post-patellar dislocation ranges from 40% to 96%, causing significant anterior knee pain, limited daily activities, and reduced athletic participation. Symptomatic patellar instability frequently correlates with trochlear dysplasia (85%), defined by the crossing sign (96%), pathological trochlear bump (>3 mm in 66%), and reduced trochlear depth (<4 mm) [5]. Initial treatment often involves nonoperative approaches, such as activity modification and physical therapy [6]. However, surgical interventions might be required in young and active individuals experiencing recurrent symptomatic dislocations to restore joint stability and enhance function. The MPFL, originating from the medial femur and averaging 56 mm in length, is crucial in surgical reconstructions aiming to address instability [7]. Advances in MPFL reconstruction techniques, graft selection, surgical fixation, and tensioning have improved functional outcomes in recent clinical studies [8]. Recognizing associated soft tissue or cartilage pathology and comprehending anatomical dissimilarity contributing to instability has led to supplementary surgical considerations, including articular cartilage procedures (e.g., chondroplasty, osteochondral allograft) or realignment surgeries (e.g. lateral retinacular release, tibial tubercle transfer, trochleoplasty). Recent studies indicate an increased rate of complications associated with patellar stabilization surgery [9]. However, there remains a need for comprehensive data on national surgical trends, utilization rates, and potential complications. Complication rates of 16.2% have been reported in MPFL reconstruction surgeries for patellar instability in young patients, with nearly half attributed to technical issues. Therefore, preoperative counseling regarding potential complications is essential for patients considering such surgeries [10].

## Case presentation

A 17-year-old female patient presented with persistent pain in her right knee for the past two years. Additionally, she reported experiencing recurrent patellar dislocations for the past two months. Notably, there was no history of a traumatic injury to the knee. Her pain began two years ago, and it gradually worsened. She started experiencing recurrent patella dislocation two months before admission, causing considerable distress and functional impairment. No specific injury or trauma was reported as the trigger for these episodes. Upon examination, she exhibited swelling and tenderness over the patella and limited range of motion, i.e., flexion and extension due to apprehension. On palpation, the patient had a positive apprehension sign. Neurovascular examination was regular.

Investigations

**Figure 1:**
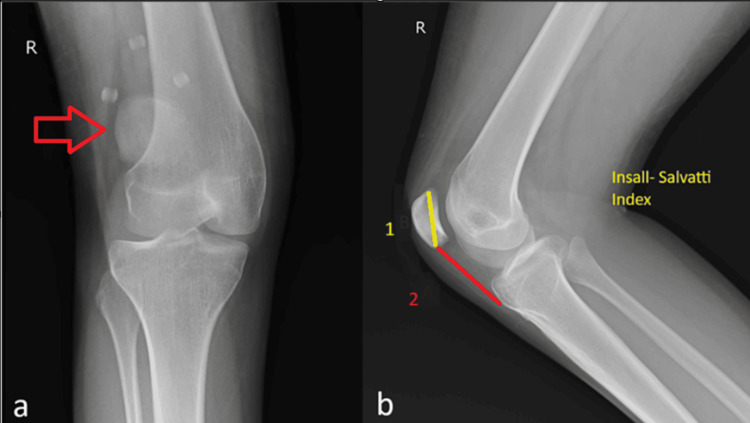
Radiography of the right knee (a) Anteroposterior view with arrow showing patella displaced laterally. (b) Lateral view showing Insall-Salvati Ratio > 1.5 indicative of patella alta, where point 1 represents the length of the patella and point 2 represents the length of the patella tendon

**Figure 2:**
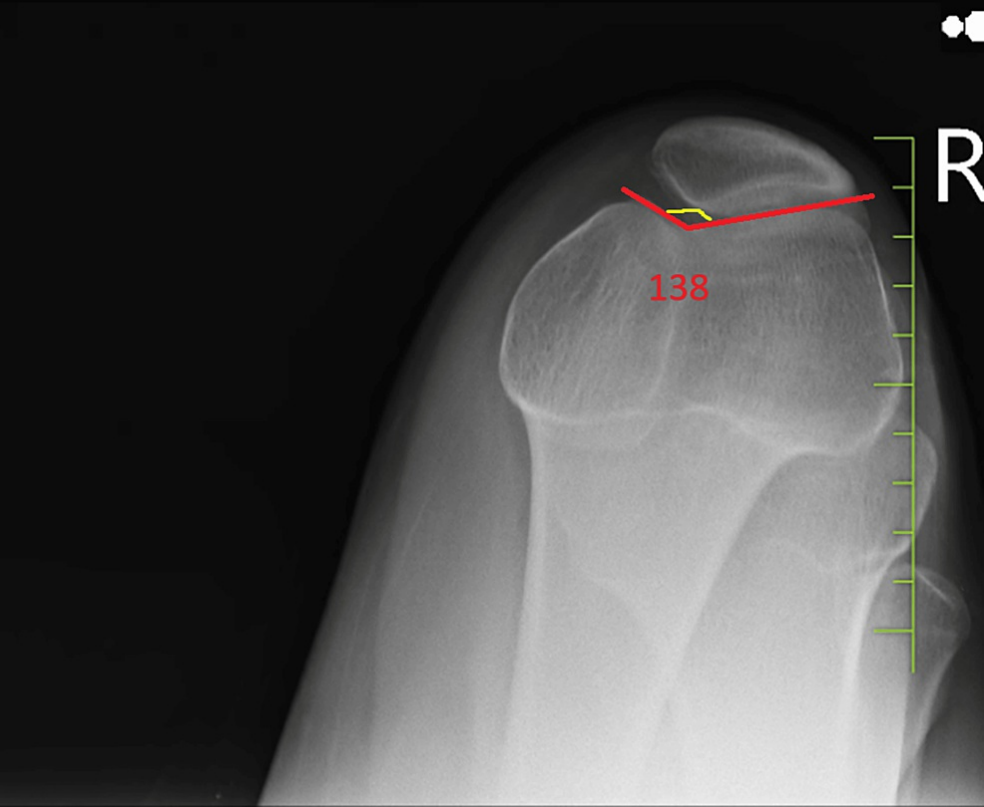
Skyline view of the right knee showing a relatively flat sulcus angle measuring 138 degrees indicative of shallow or flat trochlea

X-ray of the right knee: The X-ray revealed a laterally dislocated patella and shallow trochlear groove, confirming the clinical diagnosis, as depicted in Figures 1-2.

The measurement of the sulcus angle, which refers to the angle formed between the medial and lateral femoral facets within the trochlear groove, serves as a diagnostic tool for assessing trochlear dysplasia. In individuals with normal knee anatomy, the average sulcus angle measures around 128 degrees. However, sulcus angles exceeding 145 degrees indicate trochlear dysplasia, characterized by a shallow or flat trochlea, thereby elevating the risk of patellar dislocation [11]. An analysis of the left knee via a sunrise radiograph reveals a relatively flat sulcus angle measuring 138 degrees. Such findings are concerning, especially in patients experiencing recurrent patellar dislocations.

**Figure 3:**
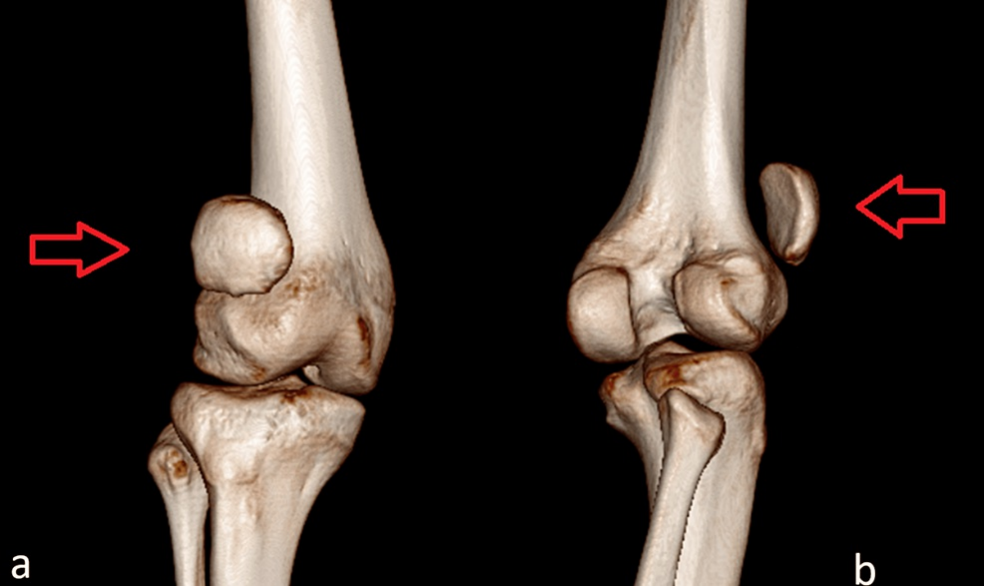
CT scan of the right knee with arrows showing superolateral displacement of the patella and posterolateral translation of the tibia with respect to the femur (a) Anterior view; (b) Posterior view

**Figure 4:**
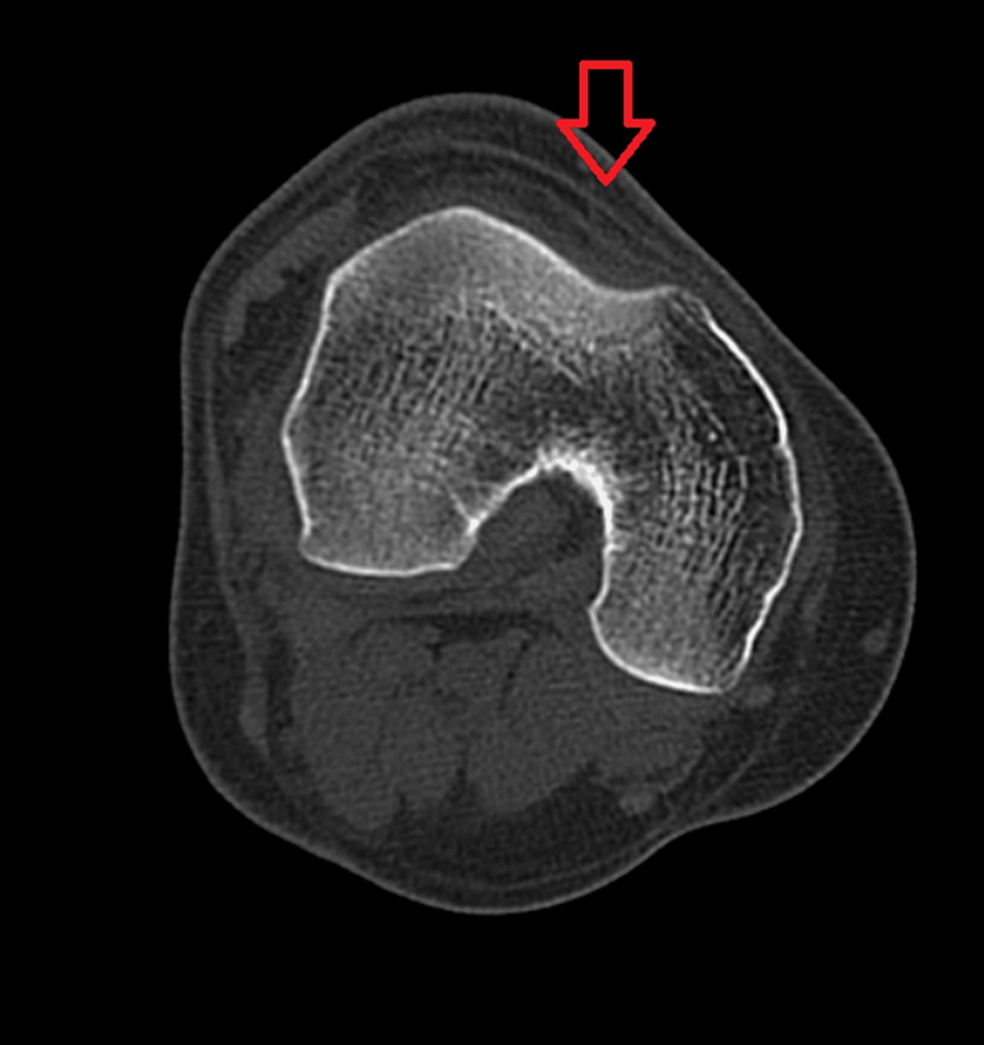
Axial section of distal femur right side showing shallow trochlear groove

CT scan of the right knee: The CT scan showed superolateral displacement of the patella indicative of patella alta and posterolateral translation of the tibia with respect to the femur, as illustrated in Figures 3-4.

**Figure 5:**
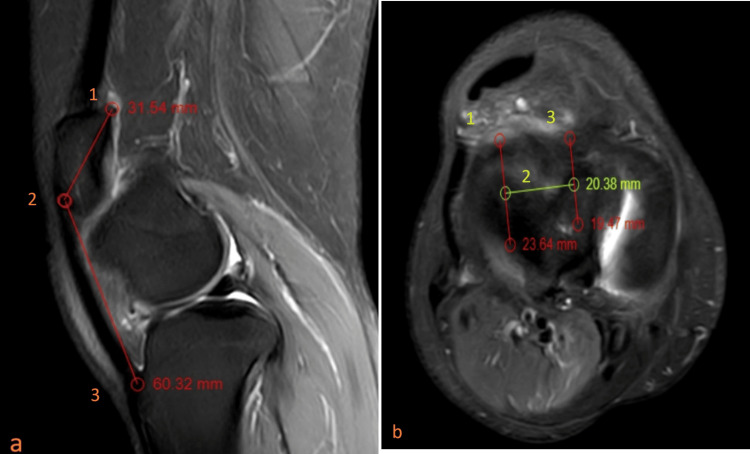
MRI of the right knee (a) Proton density fat saturation sequence in the sagittal section showing an Insall-Salvati ratio > 1.5, where points 1 to 2 represent the length of the patella bone and points 2 to 3 represent the length of the patella tendon. (b) Proton density fat saturation sequence in the axial section showing a tibial tuberosity to trochlear groove distance of 20.38 mm, where point 1 represents the trochlear groove, point 3 represents the tibial tuberosity, and point 2 represents the distance between the tibial tuberosity and trochlear groove

MRI of the right knee: The patient exhibited a complete lateral dislocation of the patella, accompanied by an increased tibial tuberosity to trochlear groove (TT-TG) distance of 20 mm. The trochlear groove is characterized by shallowness with a depth of 2 mm, and there is evidence of laxity in the medial patella retinaculum, as visualized in Figure 5.

Based on the clinical presentation and imaging findings, the patient was diagnosed with recurrent patellar dislocation associated with an increased TT-TG distance, a shallow trochlear groove, and patella alta. The patient was kept supine, accompanied by a tourniquet secured around the upper thigh. Ensuring that the bolster and tourniquet did not obstruct the arthroscope through the superolateral portal was crucial. A vertical skin incision, approximately 6-7 cm long, was made parallel to the medial side, extending from the tibial tuberosity toward the distal end. This incision allowed exposure to the tuberosity and gracilis for subsequent use in MPFL reconstruction.

The surgical procedure involved cutting the gracilis tendon and preparing its ends with a whip stitch. Special care was taken to locate and safeguard the patellar tendon attachment at the tibial tuberosity. Furthermore, the anterior portion of the tibialis anterior was raised above the tibia. An osteotomy with four cuts was then performed: two in the axial plane, one proximal oblique cut at the patellar tendon insertion, and one in the longitudinal coronal plane. This was necessary for the planning process, aiding in the accurate placement of screws. To establish the best length of the osteotomy, the coronal incision was aligned with three marks on the tuberosity, indicating the expected placements of the fixation screws. As shown on lateral radiographs, a slope in the coronal plane was avoided because it could cause the fragment to shift anteriorly or posteriorly. The osteotomy had been smooth and flat along its length, making transferring to the new location more accessible and increasing the surface area for the union. The osteotomy measured 5-6 cm. Using magnetic resonance imaging images as a guide, 8-15 mm of bone was removed at the distal end, depending on the needed distalisation.

Following the osteotomy, the fragment was retrieved from the fat pad and repositioned to ensure appropriate distal and medialisation. It was temporarily secured using a 2 mm Kirschner wire (K-wire). The fixation point was precisely in the center of the area where the fragment and tibia come into contact during medialization. Two small-fragment, totally threaded screws were inserted anteroposteriorly to maximize compression while avoiding breaking the tuberosity. Countersinking had minimized irritation caused by the tuberosity, though it may not have eliminated it. The screws were sunk deep into the fragment. For gracilis graft attachment in MPFL reconstruction, a V-shaped patellar tunnel in the coronal plane was made. The tunnel's aperture was protected from slipping by vicryl sutures. The broad patellar insertion resembled the native MPFL. Before tunnel alignment, the femoral guidewire was inserted under fluoroscopic control to check for isometry. Adequate graft tension was determined by the proper placement of the patella in the trochlear groove, which was verified using superolateral portal arthroscopic visualization. This tension was sustained while the knee was kept at around 30 degrees of flexion, and a metal interference screw, typically 7 mm in diameter, was inserted to secure the graft to the femoral side.

**Figure 6:**
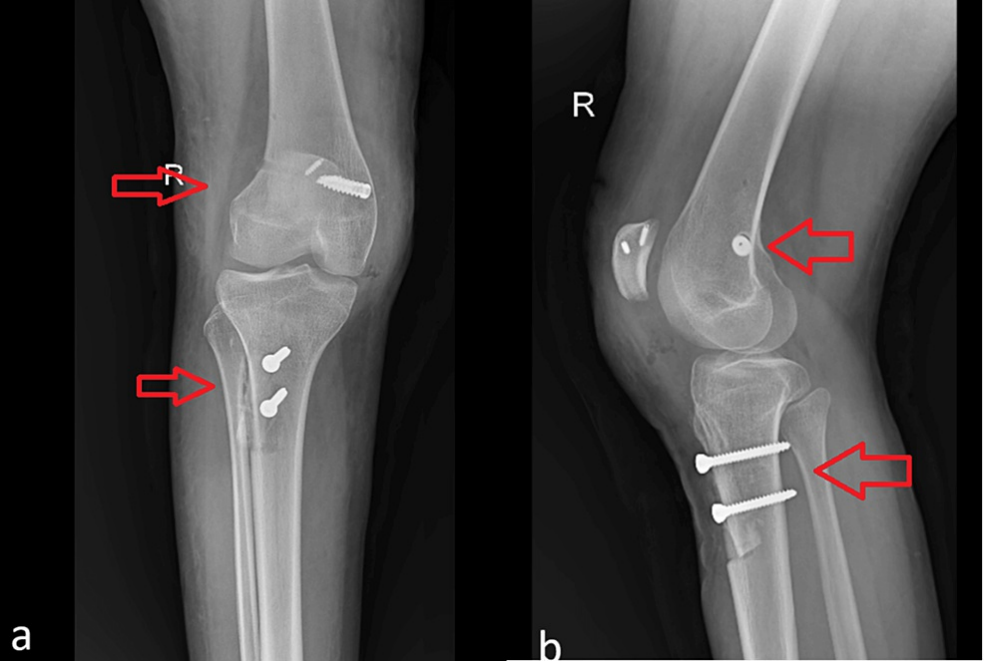
Postoperative X-ray of the right knee showing screw placement at the tibial tuberosity osteotomy site and tunnel and screw placement at the proximal pole of the patella (a) Anteroposterior view; (b) Lateral view

A postoperative X-ray was conducted. The anteroposterior view showed a centrally placed patella with implants in situ, and the lateral view displayed tibial tuberosity osteotomy (TTO) managed with screw fixation, as shown in Figure 6.

A structured physical therapy program was initiated postoperatively to strengthen the quadriceps and improve proprioception. Pain management involved prescribing nonsteroidal anti-inflammatory drugs for pain relief.

## Discussion

When combined with TTO, MPFL reconstruction gives patellar stabilization and overall pain and function benefits with low rates of recurrent instability. A recent study discovered that with no recurring subluxations or dislocations, functional outcome ratings increased significantly over at least two years [12]. Another study found that individuals who underwent MPFL reconstruction with TTO had an 83% chance of returning to sports [13]. Another study looked at the maturity of particulate juvenile articular cartilage (PJAC) grafts placed into patellar chondral lesions and found that the matured grafts matched the features of the surrounding native cartilage [14]. Because of these concerns, we advise against doing a medializing osteotomy on all persons with instability. Rather than over-medializing, any medialization should be done to establish a regular TT-TG distance.

## Conclusions

In summary, tibial tuberosity transfer with medial patellar stabilization emerges as a highly effective surgical approach to managing patellofemoral instability. This well-established procedure not only affords enduring stability but also contributes to a noteworthy enhancement in functional outcomes among individuals grappling with recurrent patellar dislocations or persistent patellofemoral pain. The commendable aspect of this surgical technique lies in its ability to deliver sustained stability over the long term while improving patients' overall quality of life. Notably, the procedural success is underscored by a favorable profile of low complication rates and a substantial degree of patient contentment. The documented outcomes suggest that this combined approach addresses the immediate concerns associated with patellofemoral instability and engenders high satisfaction among those undergoing the intervention. Such positive results bolster the case for the widespread adoption of tibial tuberosity transfer with medial patellar stabilization in the clinical management of patellofemoral instability.

Nevertheless, as with any evolving field of medical science, there remains a need for further in-depth research to refine our understanding of the optimal patient selection criteria for this specific procedure. This imperative research should also extend to rigorous comparative studies that benchmark the outcomes of tibial tuberosity transfer with medial patellar stabilization against alternative surgical techniques. Through such investigations, we can advance our knowledge, refine treatment protocols, and optimize patient care in patellofemoral instability.
